# Percutaneous Irreversible Electroporation of Unresectable Hilar Cholangiocarcinoma (Klatskin Tumor): A Case Report

**DOI:** 10.1007/s00270-015-1126-z

**Published:** 2015-05-21

**Authors:** Marleen C. A. M. Melenhorst, Hester J. Scheffer, Laurien G. P. H. Vroomen, Geert Kazemier, M. Petrousjka van den Tol, Martijn R. Meijerink

**Affiliations:** Department of Radiology and Nuclear Medicine, VU University Medical Center, de Boelelaan 1117, 1081 HV Amsterdam, The Netherlands; Department of Surgery, VU University Medical Center, de Boelelaan 1117, 1081 HV Amsterdam, The Netherlands

**Keywords:** Cholangiocarcinoma/Klatskin, Irreversible electroporation (IRE), Electroporation/methods, Tumor ablation/percutaneous, Ablation techniques/adverse effects

## Abstract

Irreversible electroporation (IRE) is a novel image-guided ablation technique that is rapidly gaining popularity in the treatment of malignant tumors located near large vessels or bile ducts. The presence of metal objects in the ablation zone, such as Wallstents, is generally considered a contraindication for IRE, because tissue heating due to power conduction may lead to thermal complications. This report describes a 66-year-old female with a Bismuth–Corlette stage IV unresectable cholangiocarcinoma with a metallic Wallstent in the common bile duct, who was safely treated with percutaneous IRE with no signs for relapse 1 year after the procedure.

## Introduction

Cholangiocarcinoma accounts for approximately 2 % of all cancer diagnoses, with an overall incidence of 1.2/100,000 individuals [1]. Two-thirds of cases occur in patients over the age of 65, with a near ten-fold increase in patients over 80 years of age [2]. Hilar cholangiocarcinomas (Klatskin tumors) account for up to 25 % of all cholangiocarcinomas [1]. Complete tumor resection (Bismuth–Corlette type I and potentially type II and III) offers the only hope for long-term survival, with 5-year survival rates ranging from 10 to 40 percent [1]. However, because most tumors become symptomatic at a late stage, less than half of the cholangiocarcinomas are resectable at time of presentation with a median survival of less than 6 months for those patients [1, 3]. No clear clinical benefit has been demonstrated for neoadjuvant and adjuvant therapies. There is no established standard palliative chemotherapeutic regimen [2].

Recently, irreversible electroporation (IRE) has been introduced as a novel technique for image-guided tumor ablation. With this technique, multiple short, high-voltage electrical pulses are delivered to the tumor tissue, which disrupt the cellular membrane and ultimately lead to cell death through apoptosis [4]. Although results are still preliminary, for pancreatic and liver tumors, the technique appears to be tolerated well and early results of efficacy are promising [5–7]. Clinical studies seem to confirm preclinical animal studies where IRE near the portal triad rarely leads to biliary complications [4].

The presence of a metallic Wallstent within the ablation zone is generally considered to be an absolute contraindication for IRE. If the electric current passes through the stent, the predicted electric field distribution may be compromised and extreme current concentration may result in stent-induced tissue heating, leading to undesirable thermal damage to sensitive structures such as intestines and bile ducts [9, 10]. Also, redistribution of the electric field may result in an unpredictable shape of the ablation zone, leading to an incomplete ablation. Commercial electroporation pulse-generators are typically limited to a 50 A maximum current and surpassing this threshold leads to a generator hard-reset for recalibration or component restabilization [8].

Despite this contraindication, uncomplicated IRE-procedures in previously stented patients with locally advanced pancreatic carcinoma have been reported (data presented at CIRSE 2013 by G. Narayanan, University of Miami, Miller School of Medicine).

Here, a case of percutaneous IRE is presented in a patient with an unresectable and growing hilar cholangiocarcinoma for which a palliative metallic Wallstent was previously placed.

### Case Presentation

**Fig. 1:**
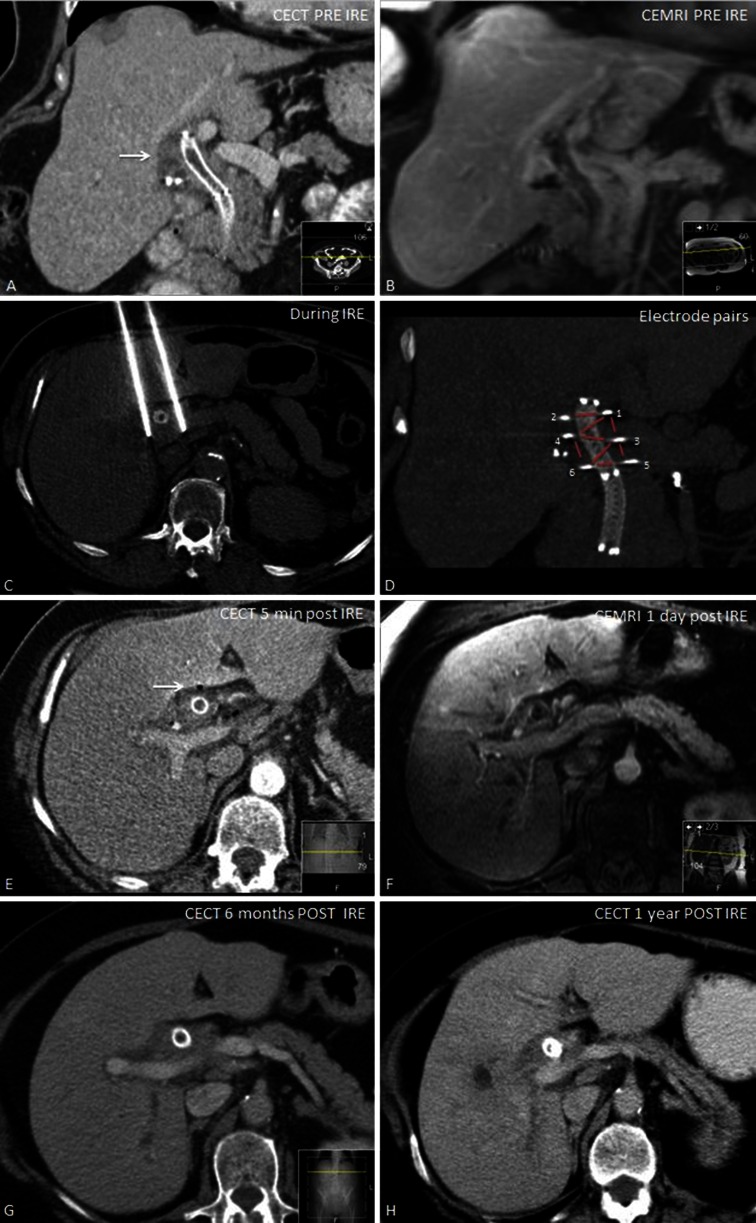
**A** Coronal ceCT image pre IRE with a hilar cholangiocarcinoma (*arrow*) surrounding a metallic Wallstent present in the common bile duct. **B** ceMRI image demonstrating an enhancing mass in the liver hilum surrounding the common bile duct. **C** Axial CT image of two electrodes placed alongside the Wallstent. **D** Coronal CT view demonstrating all six electrodes and eight electrode pairs during the ablation (*red lines*). **E** ceCT immediately after IRE demonstrating patent vessels and gas bubbles in the ablated area (*arrow*). **F** ceMRI 1 day post-IRE with no signs of complications. **G**, **H** ceCT 6 months and 1 year after IRE demonstrating no tumor progression

A 66-year-old female is presented with silent icterus (bilirubin 89 µm/L, alkaline phosphatasis U/L). Computed tomography (CT) imaging showed a 30 × 30 × 38 mm mass in the liver hilum surrounding the common and the left and right main bile ducts (Bismuth–Corlette type IV) (Fig. 1A, B). There was no sign of lymph nodal or metastatic spread and all the blood vessels were patent. An endoscopic ultrasound (EUS)-guided core needle biopsy of the lesion confirmed the diagnosis of hilar cholangioadenocarcinoma. Because of biliary obstruction, a plastic retrievable endoprosthesis was placed and renewed several times, but due to repeated congestion of the endoprosthesis, it was eventually replaced for a metallic Wallstent and bilirubin levels normalized (14 µm/L). After establishing local disease progression at 3 months, she was referred to our centre.

The patient was discussed in our weekly multidisciplinary hepatobiliary tumor board consisting of a radiologist, interventional radiologist, hepatobiliary surgeon, medical oncologist, hepato-gastroenterologist, radiotherapist, and nuclear radiologist. All potential risks and benefits of IRE near the portal triad with a metallic stent in situ were carefully outweighed against best supportive care. Since the stent did not reach into the duodenum, the risk of damaging the duodenal wall was considered low. After careful deliberation, the patient opted for IRE and gave written informed consent.

**Fig. 2:**
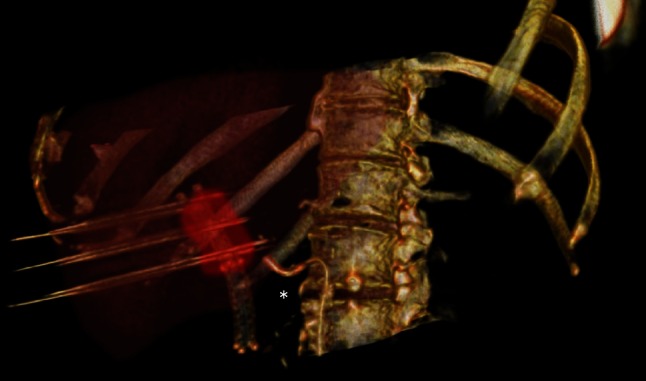
3D reconstruction with a catheter in the common hepatic artery (*asterisk*) and six electrodes placed alongside the metallic Wallstent

First, to allow repeated and real-time visualization of both the tumor and the vessels within the celiac trunk, a catheter was placed within the common hepatic artery (Fig. 2). To determine the 3-D measurements of the tumor and its proximity to other structures, a 40 mL bolus of 1:1 saline diluted contrast material was injected at 4 mL/sec with a scan delay of 7 and 30 s for the arterial and portal venous phase CT, respectively (Sensation 64 slice MDCT, Siemens, Erlangen, Germany). Size and shape, including a 5 mm margin, determined the number and configuration of the electrodes [11]. With the patient under general anesthesia in the supine position, a total of six monopolar needle electrodes (NanoKnife; AngioDynamics, Latham, New York) were placed alongside the metallic Wallstent under CT-fluoroscopy guidance, three on each side (Figs. 1C, D, 2). The active working length of the electrodes was set at 2 cm. Eight vectors for pulse delivery were chosen with a minimum and maximum interprobe distance of 1.6 and 2.5 cm (Fig. 1D). All pulses were delivered in the absolute refractory period of the heart with use of electrocardiographic synchronization (Accusync, Model 72; Milford, CT) to elude the induction of cardiac arrhythmias. To avoid generalized muscle contractions, additional rocuronium was given to achieve deep muscle paralysis. First, ten test pulses of 90 µsec were delivered for each electrode pair with a deliberately low voltage (750 V/cm) because of the presence of the Wallstent to verify the delivered current, and avoid a potential stent-induced overcurrent, which ranged between 13 and 24 A. Next, the voltage was adjusted to 1250 V/cm after which another ten test pulses were given. The delivered current appeared consistent for all electrode pairs and ranged between 24 and 40 A, so subsequently 80 treatment pulses were delivered along each vector. After removal of all six needles, contrast-enhanced CT (ceCT) demonstrated a hypodense ablation zone containing gas bubbles (Fig. 1E). The gas bubbles are presumably caused by the electrolysis of water (H_2_O) into oxygen (O_2_) and hydrogen gas (H_2_) by the electric current passing through the tissue [12]. The portal vein and the surrounding arteries were patent and the intrahepatic bile ducts appeared unremarkable.

The patient awakened uneventfully in the recovery unit and was transferred to the ward. The next day she experienced some nausea and vomiting, which was successfully treated with anti-emetics. No pain medication was required. Venous sampling showed a mild increase in transaminases (AST from 32 to 53 U/L, ALT from 26 to 52 U/L) with no signs for cholestasis (bilirubin 17 μmol/L). MRI(-DWI) 1 day post-IRE showed an edematous ablation zone surrounding the Wallstent which was present in the same location. No complications to the blood vessels or bile ducts were noticed (Fig. 1F). On the 4th day post-IRE, the patient was discharged in good clinical condition. Further recovery was uneventful. Four months later, follow-up ceCT showed no local tumor progression or metastatic disease, also no stent complications were noticed. At six, 9 months, and 1-year follow-up, ceCT still showed no local tumor progression or metastatic disease (Fig. 1G, H). She currently remains in follow-up.

## Discussion

The electrical pulses administered with IRE destabilize the cellular membranes and lead to the formation of ‘nano-pores’ in the lipid bilayer, irreversibly damaging the cell’s homeostatic mechanism [7]. IRE only affects the membranes of living cells, while preserving the extracellular matrix constituents that are responsible for the patency of major vascular and ductal structures and other vulnerable tissues [17]. Also, ablation success is not impaired by heat-sink [20]. For these reasons, the technique may be suitable to treat hilar cholangiocarcinoma.

Because hilar cholangiocarcinomas are often unresectable at presentation, in the majority of cases treatment focuses on palliation of symptoms. The preferred modality of palliation is the placement of a stent in the main biliary duct [16]. Both plastic endoprosthesis and metallic Wallstents are used for biliary drainage. Adverse effects, including cholangitis, stent occlusion, migration, perforation, and the need for reinterventions occur more frequently with plastic stents (39.3 %) than with metal stents (11.8 %) [13–15]. Therefore, in spite of the high initial cost, metallic Wallstents are considered more cost-effective for patients with tumors that are considered unsuitable for surgical resection.

The effect of metal on tissue temperature during IRE has recently been analyzed in porcine liver. Dunki-Jacobs et al. reported a mean maximum change in temperature immediately adjacent to the electrodes of 29.3 °C for ablations with metal stent in situ versus 11.3 °C for ablations without metal stents (p = 0.007) [10]. The effect of smaller metallic implants on the ablation parameters was evaluated by Neal et al. [9]. Numerical, ex vivo, and in vivo models evaluated the influence from multiple metallic brachytherapy seeds on electrical current, electric field, and temperature in tissue as well as acute histological effects [9]. There was no significant impact from the presence of these small conducting seeds on the characteristics influencing the outcome of IRE.

In this specific case, the potential thermal effect on tumor tissue surrounding the stent may actually have been beneficial in terms of ablation and oncological efficacy for three reasons. First, since tissue conductivity increases with an increase in temperature, the zone of irreversibly electroporated tissue may be larger and more complete [8]. Secondly, with an exposure length of several minutes and the before mentioned peak temperature increases surrounding the stent, the periductal tumor tissue may also have been irreversibly damaged by heat itself [12]. Thirdly, although in large part unknown, an increase in tumor temperature during ablation coincides with a stronger immune response, therefore, a potential systemic abscopal response may have been elucidated [18]. It remains unclear to what extent the metal influences the electric field, which can also lead to a less effective or less predictable ablation zone. To avoid thermal damage to the duodenal wall, the use of IRE should be discouraged if the metallic Wallstent extends into the duodenum.

In conclusion, this case report describes a technically successful percutaneous IRE procedure of a hilar cholangiocarcinoma in the presence of a metallic Wallstent in the main bile duct. However, before IRE for this indication can be implemented in the clinical setting, the biophysical effects of metallic objects within the zone of electroporation, as well as its safety and efficacy, need to be confirmed in future translational and clinical studies.
